# Role of prognostic scores in predicting in-hospital mortality and failure of non-invasive ventilation in adults with COVID-19

**DOI:** 10.1007/s11739-022-03058-x

**Published:** 2022-08-02

**Authors:** Francesca Innocenti, Cristian Lazzari, Elisa Paolucci, Anna De Paris, Alessia Lagomarsini, Federica Guerra, Patrizia Alleonato, Lisa Casalini, Michele Buggea, Francesca Caldi, Maurizio Zanobetti, Filippo Pieralli, Giulia Guazzini, Lisa Lastraioli, Fabio Luise, Alessandro Milia, Lucia Sammicheli, Lucia Maddaluni, Federico Lavorini, Riccardo Pini

**Affiliations:** 1 High-Dependency Unit, Emergency Department, Careggi University-Hospital, Lg. Brambilla 3, 50134 Florence, Italy; 2 Intermediate Care Unit, Careggi University-Hospital, Florence, Italy; 3 Department of Clinical and Experimental Medicine, University of Florence, Florence, Italy

**Keywords:** COVID-19, Respiratory failure, Non-invasive ventilation, Prognosis

## Abstract

We tested the prognostic performance of different scores for the identification of subjects with acute respiratory failure by COVID-19, at risk of in-hospital mortality and NIV failure. We conducted a retrospective study, in the Medical High-Dependency Unit of the University-Hospital Careggi. We included all subjects with COVID-19 and ARF requiring non-invasive ventilation (NIV) between March 2020 and January 2021. Clinical parameters, the HACOR score (Heart rate, Acidosis, Consciousness, Oxygenation, Respiratory Rate) and ROX index ((SpO2/FiO2)/respiratory rate) were collected 3 (-3) and 1 day (-1) before the NIV initiation, the first day of treatment (Day0) and after 1 (+1), 2 (+2), 5 (+5), 8 (+8) and 11 (+11) of treatment. The primary outcomes were in-hospital mortality and NIV failure. We included 135 subjects, mean age 69±13 years, 69% male. Patients, who needed mechanical ventilation, showed a higher HACOR score (Day0: 6 [5-7] vs 6 [6-7], p=.057; Day+2: 6 [6-6] vs 6 [4-6], p=.013) and a lower ROX index (Day0: 4.2±2.3 vs 5.1±2.3, p=.055; Day+2: 4.4±1.2.vs 5.5±1.3, p=.001) than those with successful NIV. An HACOR score >5 was more frequent among nonsurvivors (Day0: 82% vs 58%; Day2: 82% vs 48%, all p<0.01) and it was associated with in-hospital mortality (Day0: RR 5.88, 95%CI 2.01-17.22; Day2: RR 4.33, 95%CI 1.64-11.41) independent to age and Charlson index. In conclusion, in subjects treated with NIV for ARF caused by COVID19, respiratory parameters collected after the beginning of NIV allowed to identify those at risk of an adverse outcome. An HACOR score >5 was independently associated with increased mortality rate.

## Introduction

Current guidelines on the management of hypoxemic respiratory failure are very cautious about the employment of non-invasive ventilator support, which did not prove to be effective in this condition [1]. A trial can be performed in selected subjects and in adequate clinical settings, with a close monitoring to prevent respiratory deterioration. In subjects with acute respiratory failure (ARF) induced by COVID-19, guidelines recommended early endo-tracheal intubation (ETI) [2, 3]. Among non-invasive supports, a trial of treatment with High-Flow Nasal Cannulas (HFNC) was preferred over Non-Invasive Ventilation (NIV). In fact, in previous pandemics, the employment of NIV was associated with a high failure rate and could be harmful from different points of view [4–6]. It might create high transpulmonary pressures and large tidal volumes, which may aggravate the lung injury, as well as delay ETI and the beginning of invasive ventilation. Finally, NIV is an aerosol-generating procedure, which can increase the risk of the spread of the disease among healthcare workers.

On the other side, the heavy workload imposed by the pandemic on healthcare systems and the shortage of resources in the Intensive Care Units (ICUs) induced clinicians to employ non-invasive ventilation in subjects with acute respiratory failure caused by COVID-19 [7, 8]. Earliest reports showed a high failure rate, probably due to the overwhelming load of patients and inappropriate selection of those, who could be treated with NIV [7]. Thereafter, several authors showed a good rate of success [8, 9]. This finding, combined with a very high mortality rate in intubated patients, favored the employment of NIV in these subjects. Because the delay in intubation and invasive mechanical ventilation is associated with an increased mortality, the early identification of subjects at risk of failure of the non-invasive respiratory support remains a clinical challenge.

We evaluated the prognostic stratification ability of several scores, which were chosen based on the following criteria: (1) the ROX index, which includes a *P*/*F* ratio corrected by the respiratory rate, for its ease to use in daily clinical practice [10]; (2) the HACOR score, which includes Heart rate, Acidosis, Consciousness, Oxygenation, Respiratory Rate, easily available at the bedside, for its feasibility and known prognostic value [11, 12]; (3) the nomogram elaborated by Liu and coll., specifically designed for COVID patients and created to be used at the very beginning of the treatment with NIV [13].

The aim of the present study was to test the prognostic performance of different scores for the early identification of subjects with ARF by COVID-19, at high risk of in-hospital mortality and NIV failure.

## Methods

### Study design and setting

This was a retrospective study, performed in the High-Dependency Unit (ED-HDU) at the Careggi University Hospital. The ethics committee and institutional review board approved this study (NO. 17104). The Careggi University Hospital is an urban academic hospital and a tertiary care center (1300 beds, 130,000 visits in the Emergency Department per year). During the pandemic for COVID-19 disease, from March 2020, a 14-bed High Dependency Unit was created. Internists and emergency physicians, all experienced in critical care and in the management of subjects requiring NIV, managed the Unit.

### Selection of participants

We included all consecutive subjects, who were admitted to the HDU for ARF and were treated with NIV between March, 2020 and January, 2021. No dedicated respiratory therapist or technician was available in the unit during the study period. The decision to initiate NIV (Philips Respironics, Carlsbad, CA) was made by the attending physician, experienced in critical care, based on the guidelines of the American Thoracic Society [1] and the British Thoracic Society [14]. Whenever possible, a management plan was made before initiating a NIV trial about what to do in case of failure, either to intubate and mechanically ventilate the patient or to consider the NIV trial as a ‘‘ceiling’’ treatment, considering the stage of underlying disease and the patient’s wishes about advanced life support. Medical treatment for COVID-19 was based on available guidelines: hydroxychloroquine and lopinavir/ritonavir or darunavir/cobicistat were the standard of care during the first wave (March–May 2020), as well as corticosteroids during the second wave. Low Molecular Weight Heparin was employed in all patients, unless contraindicated. Therefore, we did not systematically annotate the administration of these drugs.

NIV was delivered by full-face or oro-nasal mask, which could be interchanged to avoid pressure ulcers. The Continuous Positive Airway Pressure (CPAP) ventilation was the first choice mode of ventilation. When symptoms and signs of respiratory distress or fatigue were present (increased respiratory rate and/or lactate levels), the pressure-support modality was employed. The fraction of oxygen in the gas flowing in the system was subsequently adjusted to maintain a peripheral saturation of O_2_ (SpO_2_) ≥ 94%.

We defined NIV failure by the need for ETI and invasive mechanical ventilation or death during NIV. Adhering to current guidelines, the attending physician decided to intubate the subjects. The primary outcomes were in-hospital mortality and NIV failure.

### Measurements and outcomes

Subjects were identified according to HDU admission diagnosis from electronic medical records. Demographic data, previous medical conditions and all the other parameters were extracted from a database, where we collected data of all subjects with COVID-19 admitted to our hospital, using a standardized collection template. For each patient, we collected data on the following days: three (Day-3) and one (Day-1) day before the NIV initiation, the day of the beginning of the treatment (Day 0) and one (Day1), two (Day2), five (Day5), eight (Day8) and eleven (Day11) days after NIV initiation. These time points were chosen to obtain the trend of respiratory parameters before, during and after the beginning of the treatment with NIV, to select the earliest evaluations with a good prognostic stratification ability. At every evaluation, we collected the following data: vital signs, arterial blood gas (ABG) parameters, laboratory data and ventilation modality. At every evaluation, the worst vital signs and arterial blood gas parameters were collected.

### Scores calculation

We calculated the following values: (1) paO_2_/FiO_2_ ratio; (2) alveolar-arterial (A-a) O_2_ gradient; (3) HACOR (Heart rate, Acidosis, Consciousness, Oxygenation, Respiratory rate) score; (4) ROX index and (5) Charlson index, for the evaluation of comorbidities.

The **HACOR score** was calculated as follows:

- Heart rate:
  -  < 120 b/min0
  -  ≥ 120 b/min1
- Acidosis (pH):
  -  ≥ 7.350
  - 7.30–7.342
  - 7.25–7.293
  -  < 7.254
- Consciousness (GCS):
  - 150
  - 13–142
  - 11–125
  -  ≤ 1010
- Oxygenation (P/F):
  -  ≥ 2010
  - 176–2002
  - 151–1753
  - 126–1504
  - 101–1255
  -  ≤ 1006
- Respiratory rate:
  -  ≤ 300
  - 31-351
  - 36-402
  - 41-453
  -  ≥ 464

It was analyzed as continuous value and dichotomized as ≤ or > 5, based on the original paper [11].

The **ROX index** was calculated as follows: (SpO_2_/FiO_2_)/respiratory rate.

It was analyzed as continuous value and dichotomized as < or ≥ 4.88 [10].

Finally, we calculated the **nomogram specifically validated to predict NIV failure** in subjects with ARF induced by COVID-19 based on data recorded on the first day of NIV [13]:

Total score = ([age × 0.0817] − 1.633]) + (7.819 − [0.521 × Glasgow coma scale]) + (10 − [0.385 × ROX]) + 3.844 (if use of vasopressors) + (0.359 × number of comorbidities).

The probability of NIV failure was calculated as follows:

(0.02354 × [total score]^2^) − (0.00079 × [total score]^3^) − (0.11954 × total score) + 0.13527.

Sequential Organ Failure Assessment (SOFA) score was calculated at every evaluation.

We collected data about the results of lung ultrasound performed within the first 48 h of the treatment with NIV, based on a standardized protocol. Each lung was divided in 6 zones (2 anterior, 2 lateral and 2 posterior) and each zone was cored as follows: (1) score 0: well-spaced B-lines < 3; (2) score 1: well-spaced B-lines ≥ 3; (3) score 2: multiple coalescent B-lines; (4) score 3: lung consolidation. The sum of the scores in all twelve zones yielded a final LUS score [15–17].

### Statistical analysis

Due to the retrospective design of the study, we included all the subjects who underwent NIV in the study period. However, based on the reported mortality in the original paper (21% in patients with T0 HACOR score ≤ 5 and 65% in those with HACOR score > 5) [11], the required population size was 60 patients and the study population included in the present study was more than double of the required study sample.

Continuous variables were reported as mean ± standard deviation or as median and interquartile range, and comparisons between two groups were performed with the Student t-test for normally distributed data or by Mann–Whitney’s test for non-parametric data. Categorical data were reported as counts and proportions and analyzed using contingency tables and χ2 test. A multivariate regression logistic analysis was performed to verify the independent prognostic value of the scores. To assess the ability of the nomogram model to discriminate subjects who responded to NIV, a concordance statistic (C-statistic; equal to the area under the receiver operating curve) and 95% CIs were calculated.

A *p*-value < 0.05 was considered significant. All statistical analyses were carried out using IBM SPSS software package (version 27).

## Results

We included 135 subjects, whose main anamnestic data are reported in Table 1. On Day-3 and Day-1, respectively 5/66 and 13/117 were not on O2 treatment, while 4/66 and 31/117 were treated with HFNC and all the others with conventional oxygen treatment. NIV was performed as continuous treatment in 31 subjects on Day0, 51 on Day1, 50 on Day2, 31 on Day5, 13 on Day8 and 6 on Day11. The remaining subjects alternated NIV and HFNC.

**Table 1:** Characteristics of the whole study population and based on in-hospital outcome

	All (*n* = 135)	Survivors (*n* = 100)	Non-survivors (*n* = 35)	*p*
Age (years)	69 ± 13	65 ± 13	76 ± 9	< 0.001
Male gender (%)	92 (69%)	68 (68%)	24 (69%)	1.000
**Previous medical conditions**				
Arterial hypertension (%)	80 (59%)	54 (54%)	26 (74%)	0.057
Diabetes (%)	28 (21%)	21 (21%)	7 (20%)	1.000
CAD (%)	24 (18%)	15 (15%)	9 (26%)	0.242
CHF (%)	10 (8%)	6 (6%)	4 (11%)	0.506
COPD (%)	15 (11%)	8 (8%)	7 (20%)	0.107
CKD (%)	6 (4%)	1 (1%)	5 (14%)	0.005
Neoplasia (%)	3 (2%)	2 (2%)	1 (3%)	1.000
Stroke (%)	6 (5%)	4 (4%)	2 (6%)	1.000
**Symptoms**				
Dyspnoea (%)	90 (67%)	66 (66%)	24 (69%)	1.000
Cough (%)	64 (48%)	51 (51%)	13 (37%)	0.205
Nausea (%)	2 (2%)	2 (2%)	0	0.966
Diarrhea (%)	10 (8%)	7 (7%)	3 (9%)	1.000
Syncope (%)	4 (3%)	4 (4%)	0	0.529
Fatigue (%)	23 (17%)	15 (15%)	8 (23%)	0.436
**Parameters on Day 0**				
HR (b/min)	82 ± 20	81 ± 18	83 ± 22	0.674
SBP (mmHg)	130 ± 25	130 ± 25	132 ± 26	0.643
RR (b/min)	29 ± 9	29 ± 10	29 ± 8	0.897
SpO_2_ (%)	92 ± 6	94 ± 3	91 ± 2	< 0.001
GCS	14.9 ± 0.5	14.9 ± 0.1	14.8 ± 0.8	0.182
pH	7.45 ± 0.04	7.45 ± 0.04	7.45 ± 0.05	0.962
LAC (mEq/L)	1.46 ± 1.16	1.39 ± 1.18	1.61 ± 1.11	0.313
WBC (10^–9^/L)	9.9 ± 4.9	9.7 ± 4.8	10.3 ± 5.1	0.549
Platelets (10^–9^/L)	242 ± 94	251 ± 103	225 ± 73	0.176
Creatinine (mg/dL)	1.09 ± 0.49	1.02 ± 040	1.22 ± 0.59	0.071
Bilirubin (mg/dL)	0.61 ± 0.39	0.58 ± 0.38	0.67 ± 0.42	0.366
**Treatments**				
Remdesivir (%)		16 (16%)	7 (20%)	0.606
Tocilizumab (%)	5	3 (3%)	2 (6%)	0.604
**Ventilation modality**				
*Day0 CPAP*				0.451
Continuous	13 (10%)	7 (8%)	6 (13%)	
Intermittent	57 (42%)	39 (39%)	18 (49%)	
*Day0 Bilevel*				0.261
Continuous	11 (8%)	6 (7%)	5 (11%)	
Intermittent	54 (40’%)	33 (37%)	21 (47%)	

CAD: coronary artery disease; CHF: cardiac heart failure; COPD: chronic obstructive pulmonary disease; CKD: chronic kidney disease

Mean latency between ED admission and the initiation of NIV was 2.7 ± 2.3 days and the mean duration of the treatment with NIV was 9.1 ± 5.9 days. Forty subjects underwent ETI and mechanical ventilation, with a mean latency of 6.5 ± 5.9 days after NIV initiation. Overall, the treatment with NIV was effective in 69 (51%) patients.

**Fig. 1:**
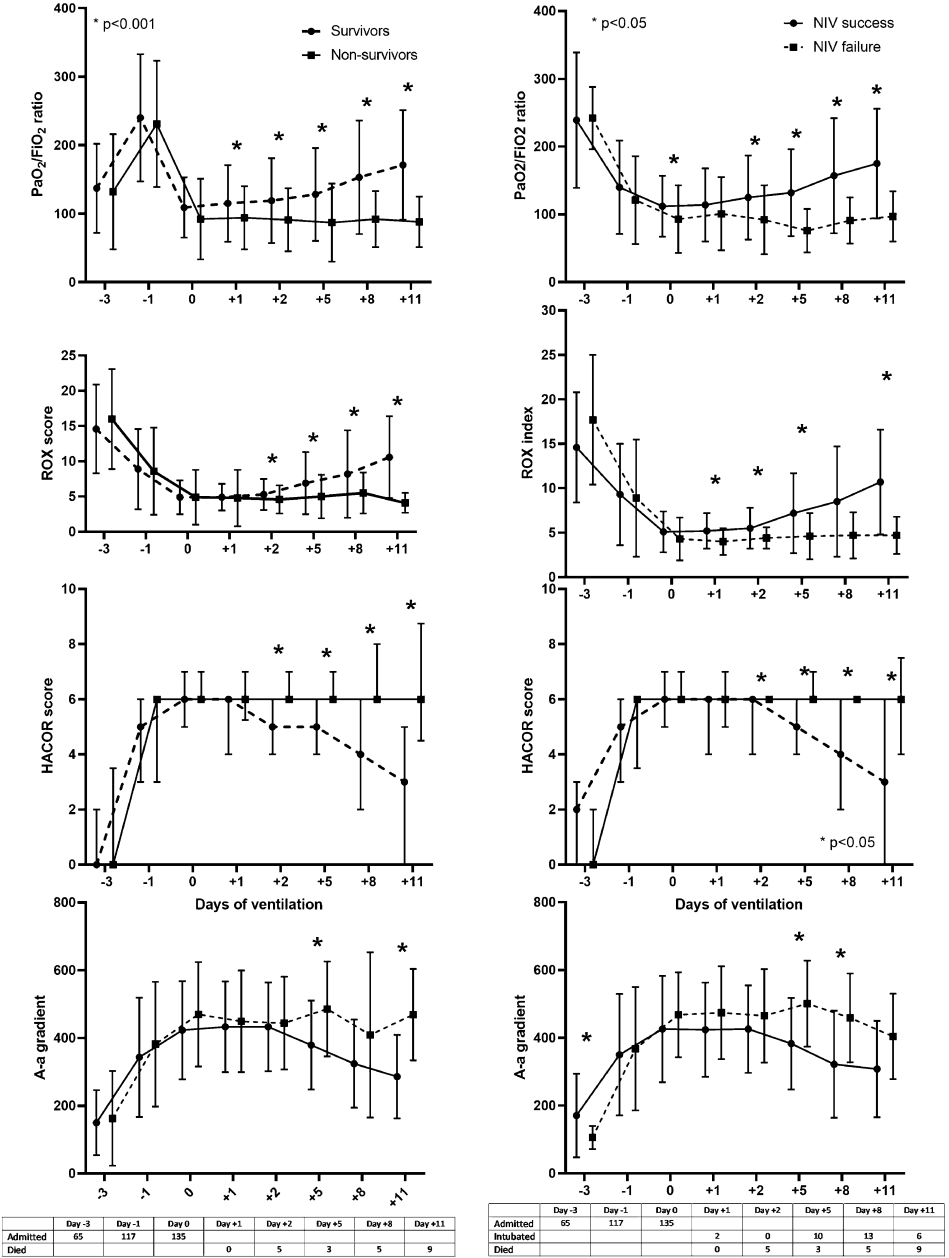
Values of *P*/*F* ratio, alveolar-arterial gradient, ROX index and HACOR score at all the evaluations in survivors and non-survivors (on the left) in subjects with successful and failed NIV (on the right). The values of the HACOR score are reported as median and interquartile range, while all the other parameters are reported as mean ± standard deviation

**Fig. 2:**
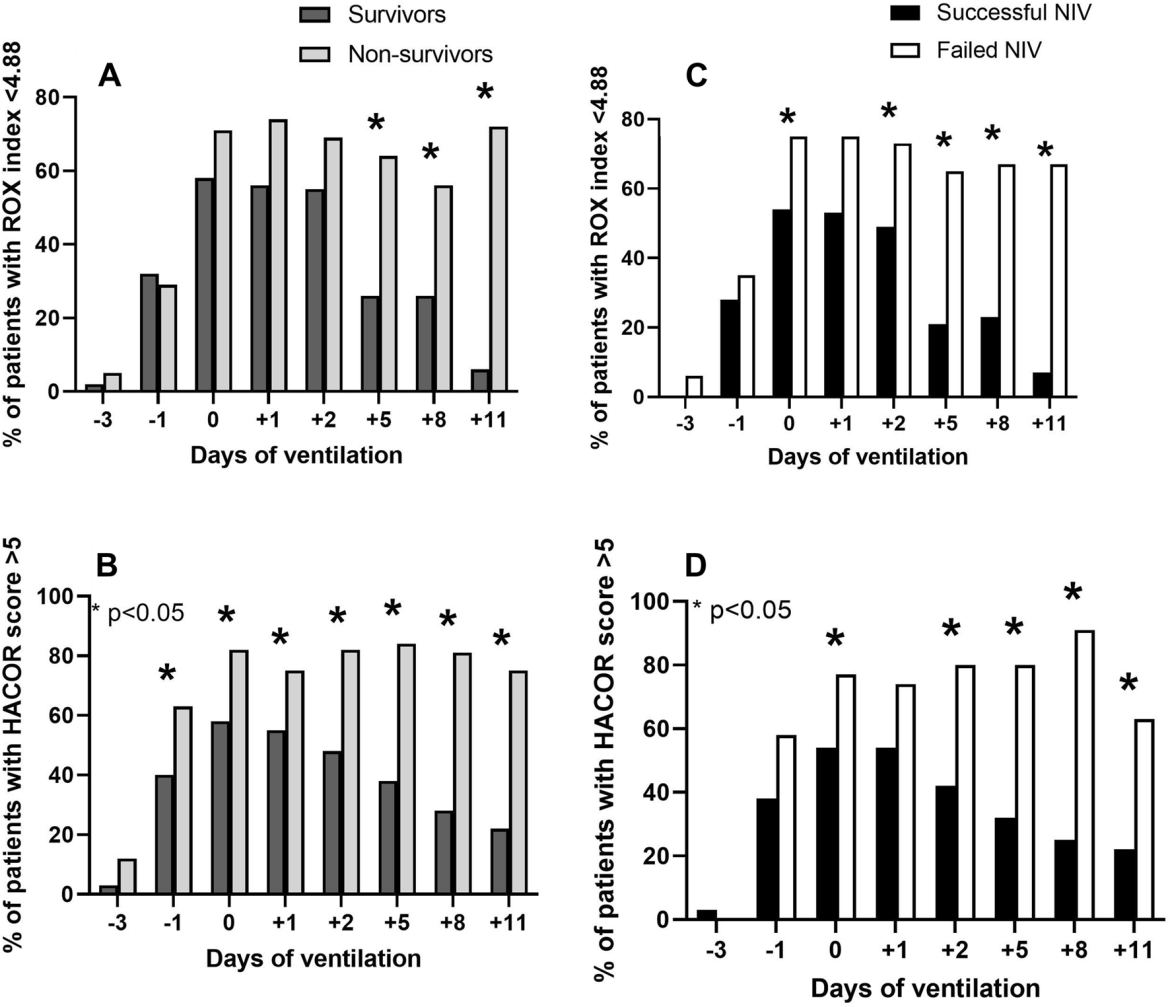
Proportion of survivors and non-survivors (**A**, **B**) and subjects with successful and failed NIV (**C**, **D**) with HACOR score > 5 and ROX index < 4.88

In the whole study population, in-hospital mortality rate was 33% (*n* = 45). Compared to subjects successfully treated with NIV, those, who underwent ETI, showed a significantly higher mortality rate (60% vs 22%, *p* < 0.001). In Table 1, we reported anamnestic and clinical parameters based on the survival status. Compared to survivors, non-survivors were significantly older, and, among comorbidities, they showed a higher prevalence of chronic kidney disease. Non-survivors showed a significantly higher Charlson index than survivors (1 [0–2] vs 0 [0–1], *p* = 0.025). We did not observe any difference regarding symptoms of presentation. Among vital signs, ABG and laboratory parameters, only SpO_2_ was significantly worse in non-survivors than in survivors. We firstly examined the trends of respiratory parameters and scores in survivors and non-survivors. In Fig. 1 (left), we reported the values of *P*/*F* ratio, alveolar-arterial gradient, ROX index and HACOR score at all the evaluations, before and after the beginning of NIV. Both survivors and non-survivors showed a rapid deterioration of respiratory parameters in the last three days before the initiation of NIV. In the same way, parameters evaluated at Day0 were similar regardless of prognosis. By Day1, survivors showed a slow improvement while subjects with an adverse prognosis continued worsening or did not show any improvement. Thereafter, we compared the distribution of dichotomized scores based on prognosis. Compared to subjects with a good outcome, a significant higher proportion of non-survivors showed a HACOR score > 5 at all the evaluation while a ROX index < 4.88 was more frequent only at the final evaluations (Fig. 2A, B). We introduced both continuous and dichotomized values of Day0 and Day2 HACOR score, ROX index and *P*/*F* ratio in multivariate regression analyses and we adjusted scores by age and Charlson index, which were significantly higher among non-survivors than among survivors. As shown in Table 2, the HACOR score, both continuous and dichotomized, showed an independent association with an increased mortality rate, as well as Day2 *P*/*F*. The dichotomized ROX index did not show a significant prognostic value. In 23 subjects, NIV was the ceiling treatment and among them the mortality rate was disproportionally high (91 vs 21%, *p* < 0.001). We repeated the aforementioned analyses after the exclusion of patients, who underwent NIV as the ceiling treatment. The results regarding the HACOR score, *P*/*F* and A-a gradient did not change (data not shown), while ROX index did not show significant differences between survivors and non-survivors.

**Table 2:** Univariate and multivariate analysis of the association between respiratory scores and in-hospital mortality

	Univariate analysis			Multivariate analysis		
	HR	95%CI	P	HR	95%CI	p
Age	1.09	1.05–1.14	< 0.001	1.09	1.04–1.13	< 0.001
Charlson Index	1.39	1.03–1.87	0.031	1.47	1.03–2.09	0.033
Day0 HACOR > 5	3.38	1.41–8.08	0.003	5.88	2.01–17.22	0.001
Day0 HACOR	1.14	0.97–1.35	0.111	–	–	–
Age	1.09	1.05–1.14	< 0.001	1.08	1.03–1.12	< 0.001
Charlson Index	1.39	1.03–1.87	0.031	–	–	–
Day2 HACOR > 5	4.89	1.95–12.24	< 0.001	4.33	1.64–11.41	0.003
Day2 HACOR	1.40	1.09–1.78	0.008	1.34	1.03–1.74	0.027
Day0 ROX < 4.88	1.80	0.83–3.88	0.134			–
Day0 ROX	0.99	0.88–1.12	0.886			–
Day2 ROX < 4.88	1.88	0.82–4.30	0.136			–
Day2 ROX	0.83	0.66–1.05	0.116			–
Day0 P/F	0.99	0.98–1.01	0.066			–
Day + 2 P/F	0.99	0.98–0.99	0.019	0.99	0.98–0.99	0.016

The values of the scores were tested by univariate analysis and then introduced in a multivariate analysis, each including the score value, either continuous or dichotomized, adjusted by the age and Charlson index.

The patients, for whom NIV was the ceiling treatment, were excluded from the analysis about the predictive parameters for ETI. Compared to subjects with successful NIV, those with failed treatment showed a similar age (71 ± 8 vs 68 ± 15 years, *p* = 0.194) and Charlson index (0.5 [0–2] vs 0 [0–1], *p* = 0.075). In Table 3, we reported vital signs, arterial blood gas and laboratory parameters, based on the effectiveness of the treatment with NIV, in the following selected evaluations: Day-1, 0, 1 and 5. Parameters collected in the days before the initiation of NIV did not show any significant difference. From Day1, parameters of respiratory function, both in terms of oxygenation and respiratory rate, as well as the scores, were significantly more compromised in subjects, in whom NIV failed. In Fig. 1 (right), we reported the values of the P/F ratio, alveolar-arterial gradient, HACOR score and ROX index at all the examinations. We confirmed the same trends evidenced in Table 3. When we considered the dichotomized values of the scores (Fig. 2C and D), a significantly higher proportion of subjects who underwent NIV failure had a HACOR score > 5 and a ROX index < 4.88. We did not perform a multivariate analysis for this outcome as subjects with successful and failed NIV showed similar age and Charlson index.

**Table 3:** Vital signs, arterial blood gas and laboratory parameters from Day -1 to Day-5 of ventilation

	Day − 1		Day 0		Day 1		Day 5	
	NIV success (*n* = 72)	NIV failure (*n* = 40)	NIV success (*n* = 72)	NIV failure (*n* = 40)	NIV success (*n* = 72)	NIV failure (*n* = 40)	NIV success (*n* = 72)	NIV failure (*n* = 40)
**Vital signs**								
HR (b/min)	84 ± 18	79 ± 14	82 ± 18	84 ± 23	76 ± 18	77 ± 19	75 ± 17	83 ± 19
SBP (mmHg)	124 ± 20	122 ± 21	132 ± 24	128 ± 26	130 ± 23	131 ± 28	133 ± 26	131 ± 26
RR (b/min)	25 ± 7	26 ± 7	29 ± 11	31 ± 10	26 ± 7	30 ± 8*	24 ± 5	28 ± 6*
SpO_2_ (%)	94.1 ± 3.6	91.4 ± 7.0*	94 ± 3.1	92.7 ± 4.7	94.5 ± 3.6	93.1 ± 4.7	94.5 ± 3.0	90.0 ± 7.4*
GCS	14.9 ± 0.12	15.0 ± 0.0	14.9 ± 0.2	14.9 ± 0.2	14.9 ± 0.9	14.9 ± 0.2	14.9 ± 0.1	15.0 ± 0.0
**ABG**								
pH	7.46 ± 0.04	7.46 ± 0.04	7.45 ± 0.04	7.46 ± 0.05	7.45 ± 0.04	7.45 ± 0.04	7.47 ± 0.03	7.48 ± 0.04
pCO_2_	36.0 ± 4.7	35.4 ± 4.9	37.6 ± 7.2	37.4 ± 8.3	38.4 ± 8.0	37.6 ± 4.9	38.7 ± 6.7	41.0 ± 4.9
LAC (mEq/L)	1.26 ± 0.56	1.31 ± 0.53	1.42 ± 1.31	1.54 ± 1.09	1.18 ± 0.82	1.27 ± 0.41	1.36 ± 0.72	1.54 ± 0.49
**Labs**								
WBC (10^–9^/L)	9.1 ± 4.2	9.2 ± 5.0	9.3 ± 4.5	10.6 ± 5.9	9.3 ± 4.6	10.3 ± 5.2	9.1 ± 3.8	13.4 ± 5.1*
Platelets (10^–9^/L)	215 ± 73	244 ± 116	240 ± 94	250 ± 106	248 ± 78	247 ± 117	303 ± 97	309 ± 118
Creatinine (mg/dL)	0.98 ± 0.36	1.18 ± 0.47*	0.98 ± 0.37	1.25 ± 0.61*	0.89 ± 0.37	1.04 ± 0.49	0.87 ± 0.85	0.92 ± 0.41
Bilirubin (mg/dL)	0.55 ± 0.22	0.72 ± 0.65	0.61 ± 0.41	0.57 ± 0.42	0.65 ± 0.43	0.61 ± 0.36	0.57 ± 0.55	0.55 ± 0.36
Ferritin (ng/mL)	752 ± 554	1297 ± 1737	967 ± 473	2079 ± 2173*	2152 ± 3400	1693 ± 1177	1135 ± 899	828 ± 636
CRP (mg/L)	105 ± 74	115 ± 73	115 ± 72	113 ± 63	81 ± 42	145 ± 84*	45 ± 45	84 ± 54*
Ddimer (ng/mL)	2099 ± 6510	1217 ± 891	2672 ± 9263	4781 ± 11,313	1958 ± 5715	7897 ± 21,556	3252 ± 6180	7899 ± 17,456
SOFA score	4 [3–5]	4 [3–5]*	4 [4–5]	5 [4–5]*	4 [3–5]	5 [3–5]	4 [3–5]	5 [4–5.75]*

HR: heart rate; SBP: systolic blood pressure; RR: respiratory rate; GCS: Glasgow Coma Scale; LAC: lactates; WBC: white blood cells; CPR: C reactive protein

^*^p < 0.05

**Fig. 3:**
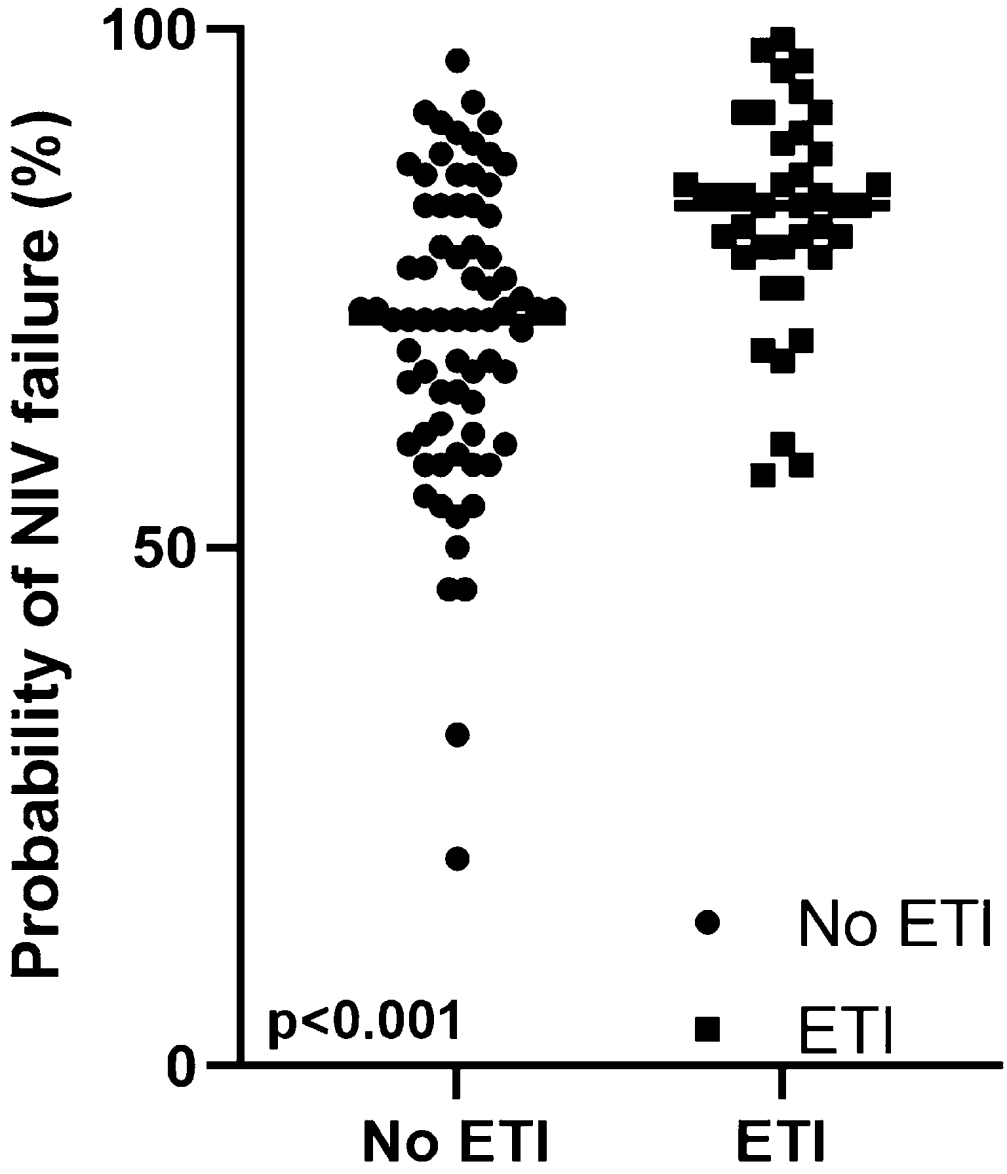
Probability of NIV failure in subjects with good and adverse prognosis

We finally calculated the nomogram to predict NIV failure with the parameters collected on Day-0 of treatment. Compared to subjects with successful NIV, the value was significantly higher in subjects with failed NIV (13.1 ± 1.1 vs 12.0 ± 1.4, *p* < 0.001), as well as the probability of NIV failure (83 ± 9% vs 71 ± 15%, *p* < 0.001), with a C-statistic 0.73. However, as shown in Fig. 3, there was a wide overlap of values of the probability of NIV failure between subjects with successful and failed NIV. We repeated the analysis with values collected at Day1. We confirmed the results obtained at Day0, with a nomogram significantly higher in intubated subjects than in those who did not (13.2 ± 1.0 vs 12.0 ± 1.5, *p* < 0.001) and a corresponding higher probability of being intubated (83 ± 9% vs 71 ± 15%, *p* < 0.001). C-statistic was also similar (0.75).

Among the 114 patients, who underwent lung ultrasound, the LUS score was similar in survivors and non-survivors (18 [14–23] vs 20.5 [14–24.3], *p* = 0.229) and in subjects with successful and failed NIV (18 [13–23] vs 20.5 [15.5–24], *p* = 0.166). Fifty-three subjects did not have any consolidation, while we observed them in 1 zone in 18 subjects (11 survivors and 7 non-survivors), in 2 zones in 22 subjects (16 survivors and 6 non-survivors), in 3 zones in 8 subjects (respectively, 4 in both subgroups) and more than 3 zones in 13 subjects (10 survivors and 3 non-survivors, *p* = 0.412).

## Discussion

In a population of patients with interstitial pneumonia caused by COVID-19, treated with NIV, we confirmed that non-invasive respiratory support was successful in about half of the subjects. We evidenced that scores calculated before the NIV initiation or at the very beginning of the treatment did not allow identifying subjects at high risk of adverse prognosis. By Day1, the values of the scores were more compromised in subjects who underwent NIV failure and in non-survivors than in those with a favorable outcome. At the earliest evaluations after the beginning of NIV, an HACOR score > 5 was independently associated with a higher mortality rate and an increased need of invasive respiratory support. The aforementioned nomogram demonstrated a fair prognostic value, with a wide overlap of values between subjects with successful and failed NIV.

Our results are encouraging as to consider the possibility to attempt a trial of NIV in these subjects. In fact, it was not possible to identify those at high risk of an adverse outcome before the beginning of the treatment with NIV. In our study population, NIV was successful in a higher proportion of subjects compared to some of the previous studies in COVID patients, despite a similar deterioration of respiratory function at the beginning of the treatment with the NIV itself [7, 18]. The key issue remains the early and accurate identification of subjects in whom NIV will fail. Parameters, which measure oxygenation, showed a severe deterioration compared to subjects with hypoxemic respiratory failure of other etiologies [19, 20]. This is consistent with the pathophysiology of pneumonia induced by COVID-19, which determines an impairment of the oxygen exchange, for a ventilation-perfusion mismatch due to the microvascular dysfunction [21, 22]. The early limited alveolar infiltrates prevent the reduction of pulmonary compliance and the consequent increased work of breathing and sensation of dyspnea, even in the presence of an increased respiratory rate [23]. Therefore, subjects tolerated severe hypoxemia without significant respiratory distress [24]. In the earliest phases of the treatment, the degree of hypoxemia was similar in subjects with good and adverse prognosis and its response to the treatment with NIV seems to have a higher prognostic value, compared to baseline parameters. Alveolar-arterial gradient, an index of oxygenation, which considers the percentage of alveolar carbon dioxide, was severely compromised, but it was significantly worse in subjects with adverse prognosis only several days after the beginning of NIV. This is consistent with the finding that pCO_2_ tended to rise by Day-5, although in a non-significant way, in those who faced NIV failure.

The ROX index, which combines the evaluation of peripheral oxygen saturation and respiratory rate, was initially conceived to predict the failure of the treatment with HFNC among patients with acute respiratory failure due to pneumonia. It could be promising for the early prognostic stratification of patients with COVID, but it became significantly different based on prognosis from Day + 1 [10]. Its limited discriminative value at the beginning of the treatment could be due to the limited accuracy of the SpO_2_ measurement in the presence of very low values (< 80%), with consequent impossibility to distinguish true and false low SpO_2_ [25, 26]. ROX index has already been employed in subjects with COVID-19 treated with HFNC as well as with NIV, to early identify those at high risk of failure, with good results [27–29] but different cut-offs have been adopted by different studies. In this population, a value < 4.88, the usual suggested cut-off, was significantly more common among those with an adverse prognosis from Day + 2, but it did not show an independent association with an increased mortality rate.

The HACOR score [11] and the nomogram elaborated by Liu and coll. [13] are both based on parameters easily obtainable at the bedside. The prognostic performance of the HACOR score has already been tested among COVID subjects, but it was tested only in the first hours after the beginning of the treatment with NIV and its prognostic value has been confirmed [30, 31]. Its good prognostic stratification ability could be ascribed to the combination of parameters expressing oxygenation status, the degree of respiratory distress and the global clinical severity. However, the novelty of the present study was the observation of the trend of the score over a long period, encompassing the days immediately before and after the beginning of the treatment with NIV. We could show that respiratory parameters collected before the initiation of NIV and the score calculated at that moment did not allow the identification of patients at risk of both NIV failure and mortality. This means that, in the presence of COVID, even patients with severe respiratory failure can undergo a trial of NIV and what really predicts an unfavorable prognosis is the lack of improvement of respiratory parameters with the ventilatory support. In fact, for the first time we demonstrated that, after the beginning of NIV, a value of HACOR score > 5 was significantly associated with an adverse outcome, independent to the age and the presence of comorbidities. Alongside, this was the first attempt to test the nomogram elaborated by Liu and coll. in a different population. Its appealing characteristics could allow us to identify subjects earlier at risk of an adverse prognosis. However, it did not cope up with expectations and, in the earliest phases of the treatment, its discriminative ability was fair.

The ultrasonographic findings did not add useful prognostic information. The values of LUS score found in this study population are consistent with previous papers [17, 32]. The absence of significant differences between subjects with favorable and adverse prognosis could find two main reasons. As for respiratory parameters, a single evaluation performed at the beginning of the treatment could not be able to distinguish between subjects who will respond to the treatment with NIV and those who will not, as, again, the response to the treatment plays a pivotal role over the baseline conditions. From an epidemiological point of view, most of the previous papers which evaluated the prognostic value of LUS score, included subjects encompassing a wide range of severity of COVID-19, from nearly asymptomatic to severe ARF. In this study population, which included only subjects with severe ARF, serial ultrasonographic evaluations could give more relevant prognostic information than a single assessment.

A separate mention deserves the disproportionally high mortality among those, who underwent NIV as ceiling treatment. We decided to include these patients in the analysis of parameters, which predicted the mortality rate, as they represented a significant proportion of subjects treated with NIV and identifying parameters for their early prognostic stratification could be useful for clinicians. Their exclusion from that analysis did not significantly modify the results, especially regarding the HACOR score. Therefore, the prognostic value of the scores we observed was not primarily due to these patients, but was valid for the whole study population.

This study has several limitations. The retrospective, single-center design may limit its applicability. We decided to observe subjects for a long period and we considered, for every evaluation, the worse parameters. We cannot exclude that different criteria could modify our results, but this choice was motivated by the need to be consistent in all subjects and during the whole period.

From the beginning of the ventilatory support, most of our patients alternated NIV and HFNC. These patients required very long treatment with NIV, so that interruptions of NIV were allowed during daytime, if they were able to maintain an SO2 > 94% without respiratory distress for brief periods. We did not systematically annotate the length of the interruptions and we cannot assess a possible prognostic weight of this modality to use NIV, but it was the norm for most of our patients. We did not systematically perform an ultrasound cardiac examination or chest CT scan and we were not able to consider the possible prognostic value of the presence of new-onset right ventricular systolic dysfunction or thromboembolic events [33]. To what extent these alterations affect the response to the treatment with NIV has not been definitively evaluated and needs to be explored in future studies.

### Conclusion

We observed that the treatment with NIV was successful in a relevant proportion of subjects. This could be a support to perform a trial of NIV in all the subjects with COVID-19, who do not have clear contraindication to this kind of support. The assessment after 24–48 h of treatment with NIV gave the best prognostic information in these subjects, while the evaluations before and concomitant with the NIV initiation did not allow the identification of subjects at high risk of adverse prognosis. An HACOR score > 5 after the initiation of NIV was independently associated with an increased mortality rate and a high prevalence of NIV failure, independently to age and the presence of previous medical conditions.

## Data Availability

Not applicable.

## Data Availability Material

The datasets generated during and/or analyzed during the current study are available from the corresponding author on reasonable request.
